# Literary Periodicals

**Published:** 1872-03

**Authors:** 


### LITERARY PERIODICALS.

The *Southern Magazine*, for March, is filled with interesting matter. The literary character of this Magazineis solid, and free from the sensational type of literature of the day. Besides being devoted to general science, each number contains literature of a lighter character, from the most gifted of Southern and other authors. Subscription price $4.00. Address Murdoch, Browne & Hill, 166 Baltimore St., Baltimore, Md.

*Hearth and Home.*—This is our favorite illustrated weekly, and is certainly entitled to rank in the front, if not at the head, of weekly illustrated papers. The engravings are always interesting, instructive, and well executed, while the moral tone, and general literature is not excelled by any periodical in the country. It is a weekly literary treat for the family, which all should secure. Address Orange Judd & Co., 245 Broadway, New York. Price, $3.00 per annum.

